# Influence of Network Size on Adversarial Decisions in a Deception Game Involving Honeypots

**DOI:** 10.3389/fpsyg.2020.535803

**Published:** 2020-09-25

**Authors:** Harsh Katakwar, Palvi Aggarwal, Zahid Maqbool, Varun Dutt

**Affiliations:** ^1^Applied Cognitive Science Laboratory, Indian Institute of Technology Mandi, Kamand, India; ^2^Dynamic Decision Making Laboratory, Carnegie Mellon University, Pittsburgh, PA, United States

**Keywords:** honeypot, cybersecurity, cyber deception, deception game, adversary, defender, probes, attacks

## Abstract

Deception via honeypots, computers that pretend to be real, may provide effective ways of countering cyberattacks in computer networks. Although prior research has investigated the effectiveness of timing and amount of deception via deception-based games, it is unclear as to how the size of the network (i.e., the number of computer systems in the network) influences adversarial decisions. In this research, using a deception game (DG), we evaluate the influence of network size on adversary’s cyberattack decisions. The DG has two sequential stages, probe and attack, and it is defined as DG (n,k, γ), where n is the number of servers, k is the number of honeypots, and γ is the number of probes that the adversary makes before attacking the network. In the probe stage, participants may probe a few web servers or may not probe the network. In the attack stage, participants may attack any one of the web servers or decide not to attack the network. In a laboratory experiment, participants were randomly assigned to a repeated DG across three different between-subject conditions: small (20 participants), medium (20 participants), and large (20 participants). The small, medium, and large conditions used DG (2, 1, 1), DG (6, 3, 3), and DG (12, 6, 6) games, respectively (thus, the proportion of honeypots was kept constant at 50% in all three conditions). Results revealed that in the small network, the proportions of honeypot and no-attack actions were 0.20 and 0.52, whereas in the medium (large) network, the proportions of honeypot and no-attack actions were 0.50 (0.50) and 0.06 (0.03), respectively. There was also an effect of probing actions on attack actions across all three network sizes. We highlight the implications of our results for networks of different sizes involving deception via honeypots.

## Introduction

Cyberattacks, organized attempts to disable computers, steal data, or compromise websites, have been steadily increasing (Trustwave, 2019). For example, there was a rise of 56% in detected web-based cyberattacks on enterprise networks in 2018 compared to 2017 (Symantec, 2019). Some of the detected web-based attacks in 2018 included SQL injection, path traversal, and cross-site scripting, which accounted for more than 50% of cyberattacks on corporate resources (PosTech, 2020).

Due to the prevalence of different kinds of cyberattacks and the associated cyber-defense costs (Hope, 2020), one may need to develop and evaluate technologies that provide security against cyberattacks (Sayegh, 2020). Currently, there are a few solutions that could help us in countering attacks (Matthews, 2019). For example, networks could contain intrusion detection systems (IDSs), which warn defenders about potential cyberattacks (Bace and Mell, 2001; Aggarwal et al., 2018; Aggarwal and Dutt, 2020). Although robust, IDSs may suffer from false alarms (indicating a cyber-threat when one is not present) and misses (missing to show a cyber-threat when it is present) (Mell et al., 2003). These false alarms and misses could lead to loss of revenue and significant damages to cyberinfrastructure, respectively (Shang, 2018a). Prior research has also proposed that hybrid censoring and filtering strategies may enable bounded non-rational network agents to reach consensus behavior (Shang, 2018b, 2019). Overall, such consensus could be useful in detecting cyberattacks before they become damaging (Shang, 2018b).

Beyond IDSs and filtering strategies, another solution that has been shown to be effective against cyberattacks is deception (Cohen, 2006; Aggarwal et al., 2016a; Almeshekah and Spafford, 2016; Dutt et al., 2016). In fact, deception via honeypots (systems that pretend to be real) has been a prominent technique for the detection, prevention, and response to cyberattacks (Garg and Grosu, 2007; Rowe and Custy, 2007; Heckman et al., 2013; Aggarwal et al., 2016a,b; Almeshekah and Spafford, 2016). In the real world, such honeypots may be created via port hardening or by putting fake content in computer systems (Shimeall and Spring, 2013). Deception via honeypots has also been used in cutting-edge technologies like the Internet of things (IoT) to defend against modern cyberattacks (La et al., 2016).

Some researchers have proposed games to study the role of deception in cybersecurity mathematically (Garg and Grosu, 2007; Kiekintveld et al., 2015; Aggarwal et al., 2017). However, more recently, researchers have investigated human decisions in the presence of deception in abstract Stackelberg security games (Cranford et al., 2018) as well as applied games like HackIT (Aggarwal et al., 2019, 2020). Here, researchers have relied upon behavioral game theory (Camerer, 2003) and cognitive theories like instance-based learning theory (IBLT) (Gonzalez et al., 2003; Gonzalez and Dutt, 2011, 2012; Dutt and Gonzalez, 2012; Dutt et al., 2013) to understand human decisions in different cyberattack scenarios (Aggarwal et al., 2020).

Human decisions in different cyberattack scenarios may be influenced by a host of different factors, including variety and complexity of cyberattacks, network topology, and the number and diversity of zero-day vulnerabilities (Garcia-Teodoro et al., 2009; Wang et al., 2010; Lenin et al., 2014). One factor that has been less investigated and that is likely to influence human decisions in cyberattack scenarios is the network size (i.e., the number of computer systems in the network; Bagchi and Tang, 2004; Wang et al., 2010). For example, Bagchi and Tang (2004) demonstrated via computational modeling that network size was an influencing factor in different kinds of cyberattacks. Similarly, as per Bagchi and Tang (2004) and Wang et al. (2010), as the size of the network increases, one expects growth in the proportion of cyberattacks. Although prior research has investigated the influence of network size on cyberattacks via computational modeling, very little is known on how the size of the network influences human adversarial decisions in games involving deception.

Thus, the primary objective of this research is to understand the influence of network size on human adversarial decisions in games involving deception. Specifically, we develop a novel cybersecurity game involving deception via honeypots, and we vary the number of computer systems in a simulated network in the game across different experimental conditions. In the deception game (DG), adversaries can first probe some of the computer systems and then decide what systems to attack for real. In a network of different sizes, the proportion of honeypots remains constant. The outcomes of this research may help cybersecurity professionals in understanding the robustness of the honeypot network architectures of varying sizes against modern cyberattacks.

In what follows, first, we detail a DG and how the network size was varied in this game. Next, we state our expectations on the influence of network size on decisions in DG using IBLT. Furthermore, we test these expectations in an experiment involving human participants. Finally, we evaluate the results from the experiment and highlight their implications for using deception in the real world.

## The DG

The DG is a sequential, single-player game, i.e., a game between an adversary and a network (Garg and Grosu, 2007; Aggarwal et al., 2016a,b). The game is formally denoted as DG (n,k, γ), where n is the total number of web servers, k is the number of honeypots, and γ is the number of probes after which the adversary makes his final decision to attack the network or not and γ should be less than or equal to k (Garg and Grosu, 2007). There are two kinds of web servers in the game, regular and honeypot. Regular web servers are the real web servers, which contain valuable information, whereas honeypots are fake servers, which pretend to be regular with the aim of trapping adversaries to extract meaningful information. The objective of the adversary is to attack the regular web server and gain maximum points.

The game is played for multiple rounds. In each round of this game, we have two stages, the probe stage and attack stage. In the probe stage, an adversary could probe web servers multiple times. Probing means clicking on the button which denotes a web server in the game’s interface. For each probe, the adversary gets a response from the system about the system being a regular (real) web server or a honeypot (fake) web server. This feedback may or may not be accurate depending on the absence or presence of deception, respectively. Thus, this scenario may not allow the adversary to learn across a number of rounds of play. Furthermore, the game dynamics may likely mimic the real world, where adversaries may only have limited information about the nature of the infrastructure they are trying to compromise. Overall, the purpose of deception is to fool the adversary by making her believe in false information about the state of the servers. If deception is present in a round, then the network response is opposite the actual state of web servers. Thus, if the adversary probes a regular web server, then the network’s response is “honeypot,” and if the adversary probes a honeypot, then the network’s response is “regular.” If deception is not present in a round, then the network’s response will be the same as the actual state of web servers. Thus, if the adversary probes a regular web server, the network’s response is “regular,” and if the adversary probes a honeypot web server, the network’s response is “honeypot.” In the probe stage, the adversary has an additional option not to probe any web server. Deception and unreliability in feedback of the probe stage might increase no-attack actions, as the unreliable feedback of the probe stage will likely make the adversary avoid risk for regular/honeypot attack actions.

Once the adversary has made γ number of independent probes (or decides not to probe any web server), the game enters the attack stage. In the attack stage, the adversary decides to attack one of the web servers once. Attacking means clicking on the button which denotes a web server. The adversary may also decide not to attack any web server in the attack stage. Based upon the decisions made during the probe and attack stages, the adversary may win or lose points. Table 1 shows the payoff matrix for the adversary based upon the decisions in the probe and attack stages in the DG.

**TABLE 1:** Adversary’s payoff during the probe stage and attack stage in the DG.

Stage	Adversary’s Action	Adversary’s Payoff
Probe	Regular web server	+5 points
	Honeypot web server	−5 points
	Do not probe	0 points
Attack	Regular Web server	+10 points
	Honeypot web server	−10 points
	Do not attack	0 points

As shown in Table 1, in each round, if the adversary probes/attacks a regular web server, then the adversary is awarded positive points. If the adversary probes/attacks a honeypot web server, then the adversary is awarded negative points. If the adversary does not probe/attack any web server in any of the rounds, he neither loses nor gains any points. Thus, if the adversary probes a regular web server, he gains +5 points, whereas on probing a honeypot web server, he loses -5 points. If the adversary attacks a regular web server, he gains +10 points, whereas he loses -10 points on probing a honeypot web server. After completion of the attack stage, the total score of a round is calculated; and at the end of the multiple rounds, the cumulative score is calculated. The values of the payoff in Table 1 were motivated from prior literature (Aggarwal et al., 2016a,b).

## Influence of Network Size on Adversary’s Decision

In our experiment, there were three different versions of the DG to simulate networks of different sizes. Motivated from networks in the real world, the versions of the game included DG (2, 1, 1) (small), DG (6, 3, 3) (medium), and DG (12, 6, 6) (large). We kept the proportion of honeypots to the total number of web servers constant (at 50%) across the three versions of the game. Also, the number and sequence of deception and non-deception rounds were kept the same for all three versions of the DG.

Though the proportion of honeypots is the same across all three network sizes, we expect adversaries to probe and attack regular and honeypot web servers much less in the small-sized network compared to medium- or large-sized networks. One could explain this expectation based upon cognitive theories like IBLT (Gonzalez et al., 2003; Gonzalez and Dutt, 2011, 2012; Dutt and Gonzalez, 2012; Dutt et al., 2013). As per IBLT, human decisions may be driven by the exploration of available options during information search (probing) and their exploitation during real decisions (attack). Decision making during different probe and attack stages will be likely determined in a bounded-rational manner by reliance on recency and frequency of decision and their outcomes (i.e., human decisions will be driven by forgetting of distant instances and recall of only recent instances). When the network size is small, the decisions during probe and attack stages in DG involve a choice between two web servers, where one of them is a honeypot. Given the smaller number of web servers, it may be easier for bounded-rational decision makers to recall the mapping of web servers being regular or honeypot from memory. That is because fewer instances will be created in memory corresponding to the different web servers, and their activations will be high in memory due to smaller delays in their exploration during probing. However, in the medium- and large-sized networks, due to the presence of multiple web servers, bounded-rational decision makers may not be able to easily recall the mapping of web servers as regular or honeypot from memory. That is because multiple instances, one per web server, will be created in memory, and the activation of these instances will likely not be high in memory due to the long delays in their exploration during probing. Overall, the difficulty in the recall of distant instances in medium- and large-sized networks may cause more exploration of web servers during the probe stage and the attack stage in these configurations compared to that in the small-sized network. Thus, based upon IBLT, one expects that the proportion of probe and attack actions on regular and honeypot web servers will be more in medium- and large-sized networks compared to the proportion of probe and attack actions in the small-sized network. Furthermore, as instances corresponding to no-probe and no-attack actions will be more activated in memory in the small-sized network compared to medium- and large-sized networks, we expect a larger proportion of these no-probe and no-attack actions in the small-sized network compared to medium- and large-sized networks. That is because no-probe and no-attack instances in memory will be easier to recall in a small-sized network compared to medium-sized or large-sized networks. Next, we test these expectations based upon IBLT in an experiment involving human decision makers making decisions in DG.

## Experiment

In this section, we detail the experiment we carried out with human participants performing as adversaries across all rounds in the DG. The game was used to calculate the effectiveness of honeypots in different-sized networks.

### Methods

#### Experiment Design

Participants performing as an adversary (“hacker”) were randomly assigned to one of three between-subjects network size conditions (*N* = 20 participants per condition): DG (2, 1, 1) (small), DG (6, 3, 3) (medium), and DG (12, 6, 6) (large). Each condition in DG was 29 rounds long, where there were 14 deception rounds and 15 non-deception rounds (participants did not know what rounds were deception rounds and what rounds were non-deception rounds). The sequence of the deception and non-deception rounds was randomized once and then kept the same across all three conditions (see the Supplementary Material for the sequence of deception and non-deception rounds). In a round, the assignment of honeypots and regular web servers to buttons was done randomly. In the small network, the DG involved two web servers, where one of them was randomly assigned as a honeypot, and the adversary could probe one of the web servers in the probe stage (the adversary may also decide not to probe any of the web servers). In the medium network, the DG involved six web servers, where three web servers were randomly selected to be honeypots, and the adversary could probe web servers three times in the probe stage (the adversary may also decide not to probe any of the web servers). In the large network, the DG involved 12 web servers, where six web servers were randomly selected to be honeypots, and the adversary could probe the web servers six times in the probe stage (the adversary may also decide not to probe any of the web servers). Across all network sizes, after completion of the probe stage, the adversary entered the attack stage. If the adversary decided not to probe a web server anytime during the probe stage, then the probe stage ended, and the adversary entered the attack stage. In the attack stage, the adversary either decided to attack one of the web servers or decided not to attack any of them. In each condition, dependent measures included regular probe/attack proportions, honeypot probe/attack proportions, and no-web server probe/attack proportions. For computing these proportions, each regular probe/attack action by a participant in a round was coded as rp/ra, each honeypot probe/attack action by a participant in a round was coded as hp/ha, and no-web server probe/attack action was coded as np/na. Later, we computed the proportions as rp/Tp, ra/Ta, hp/Tp, ha/Ta, np/Tp, and na/Ta, where Tp and Ta were the total number of decisions during probe and attack stages, respectively, in a condition. Later, these proportions were averaged across all participants in a condition.

#### Stimuli

**FIGURE 1:**
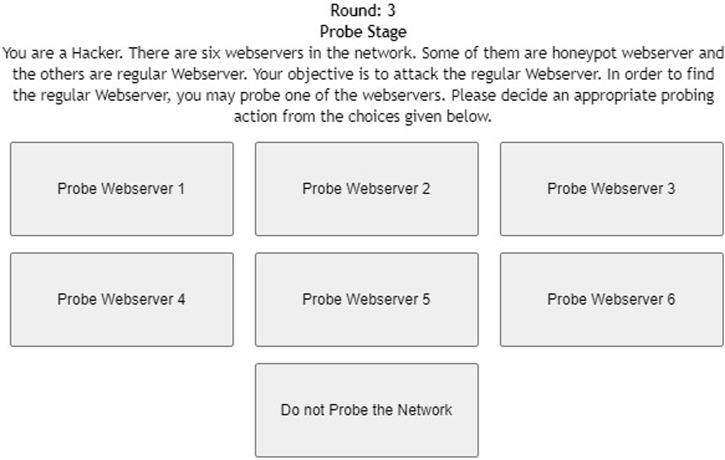
Probe stage of the deception game with six web servers.

**FIGURE 2:**
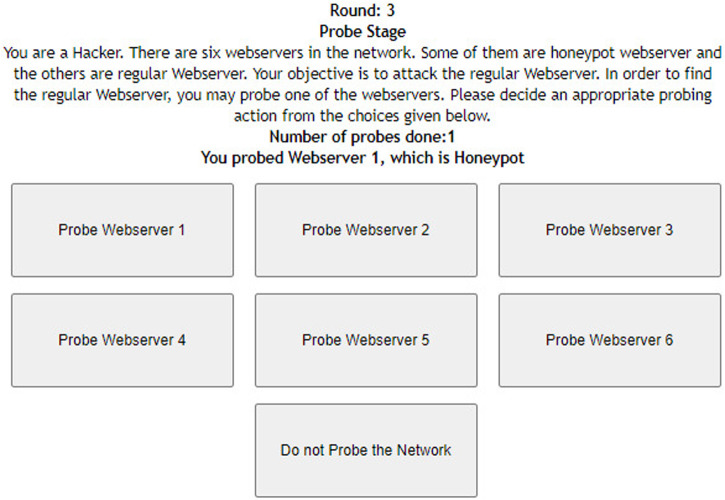
Probe stage of the deception game with six web servers after the participant probes for the first time.

**FIGURE 3:**
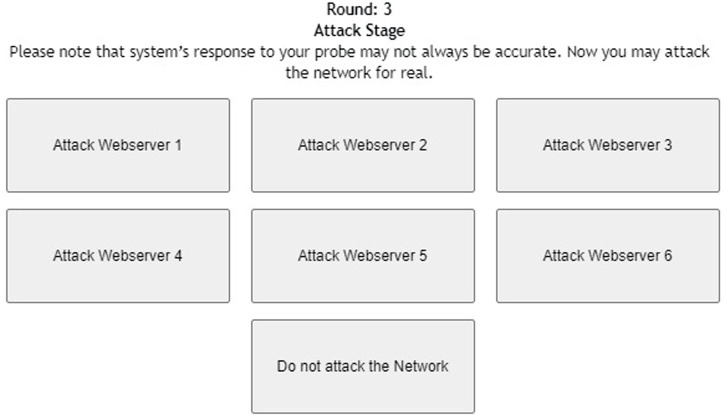
Attack stage of the deception game with six web servers.

**FIGURE 4:**
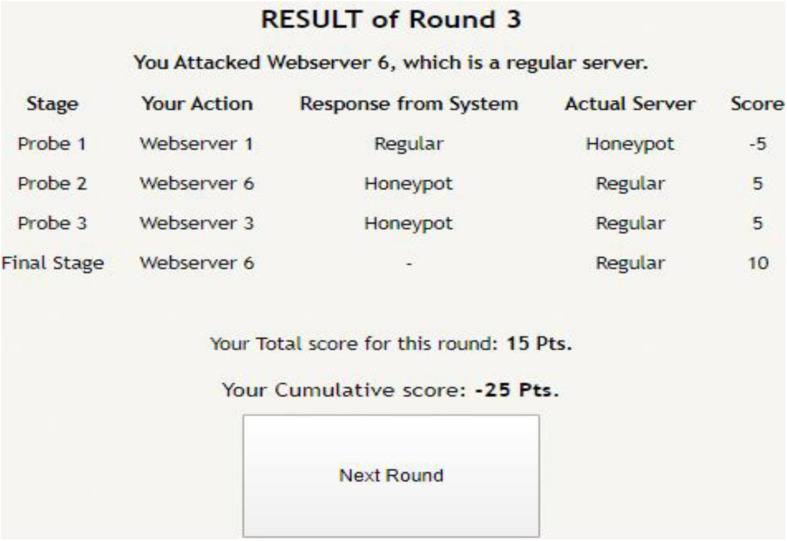
Result of a completed round, where a participant gets to know his score based upon his actions in the probe and attack stages.

Figure 1 shows the interface shown to participants in the probe stage of the DG with six web servers. As shown in the figure, participants were informed about the task with short instructions regarding the different types of web servers. Once the participant probed one of the web servers by clicking the corresponding button, she received the response from the web server (see Figure 2). Once the participant had probed for a fixed number of times, she proceeded to the attack stage (see Figure 3). After attacking one of the web servers in the network, the participant’s score was calculated for the round based on his actions in the probe and attack stages (see Figure 4).

#### Participants

This study was conducted after approval of the Ethics Committee at the Indian Institute of Technology Mandi (IITM/DST-ICPS/VD/251) with written consent from all participants. Participation was voluntary, and all participants gave written consent before starting their study. Participants were anonymously recruited for the cybersecurity study through the Amazon Mechanical Turk, a crowdsourcing website (Mason and Suri, 2012). Eighty-six percent of participants were male, and the rest were females. The age of participants ranged between 19 and 48 years (median = 31 years, mean = 32, and standard deviation = 6 years). Around 92% of participants possessed a college degree, while the remaining 8% were currently pursuing a college degree. Also, 60% of the participants had science, technology, engineering, and mathematics as a major. Participants were paid a participation fee INR 50 (USD 0.7) after they completed their study. The top three scorers of the game were chosen for the lucky draw contest, and one of these participants was randomly selected for a gift voucher of INR 500 (USD 7.14). The score was computed based upon points earned in the game during the probe and attack stages across 29 rounds.

#### Procedure

Participants performing as adversaries were given instructions about their goal in DG. Participants were told that there might be deception present in DG with both regular and honeypot servers; however, participants were not told which exact web servers were regular and which were honeypots. Participants were asked to maximize their score across several rounds involving the probe and attack stages in DG, but the endpoint in the study was not disclosed to participants. Each round has two stages: the probe stage and the attack stage. An adversary could probe multiple web servers in the DG for medium and large networks, whereas she could probe only one web server in a small network. In all network size conditions, adversaries could attack only one of the web servers in the attack stage. Once the study was completed, participants were thanked and paid for their participation. A copy of the instructions from one of the conditions is provided as Supplementary Material.

#### Data Analyses

We used analysis of variance (ANOVA), a statistical technique, to test differences between two or more means across different network size conditions (Field, 2013). Also, as sample sizes were equal across different conditions, we used the Tukey *post hoc* test (Field, 2013). The alpha level or the *p*-value (the probability of rejecting the null hypothesis when it is true) was set at 0.05, and power (the probability of rejecting the null hypothesis when it is false) was set at 0.80. We performed one-way ANOVAs to investigate the influence of network size on regular attack, honeypot attack, and no-attack decisions during the probe and attack stages. Also, we performed two-way mixed-factorial ANOVAs with network size as a between-subjects factor and sequential probe-attack trials as a within-subjects factor. Based upon the Q–Q plots (between expected quantiles and normal quantiles), different dependent variables (regular probe/attack decisions, honeypot probe/attack decisions, and no-probe/attack decisions) were found to be normally distributed. Similarly, Levene’s test showed that the variances were homogeneous for different decisions during both the probe and attack stages: honeypot web server probe [*F*(2,57) = 0.641, *p* = 0.53], regular web server probe [*F*(2,57) = 1.22, *p* = 0.30], no web server probe [*F*(2,57) = 0.382, *p* = 0.68], regular web server attack [*F*(2,57) = 2.11, *p* = 0.13], honeypot web server attack [*F*(2,57) = 1.19, *p* = 0.31], and no web server attack [*F*(2,57) = 3.70, *p* = 0.07].

## Results

### Descriptive Statistics

In our experiment, we had three different dependent variables in the probe and attack stages in the DG. In the probe stage, we had a regular web server probe, honeypot web server probe, and no web server probe. Similarly, in the attack stage, we had a honeypot web server attack, regular web server attack, and no web server attack. Table 2 describes the descriptive statistics for different dependent variables in the experiment across all conditions.

**TABLE 2:** Descriptive statistics for different dependent variables in the experiment.

Stage	Dependent Variable	Mean	Std. Deviation	Minimum	Maximum
Probe	Honeypot web server probe	0.38	0.18	0.03	0.56
	Regular web server probe	0.39	0.13	0.06	0.57
	No web server probe	0.23	0.27	0.00	0.91
Attack	Honeypot web server attack	0.40	0.18	0.07	0.66
	Regular web server attack	0.40	0.12	0.17	0.69
	No web server attack	0.20	0.25	0.00	0.69

### Influence of Network Size on Decisions During the Probe Stage

**FIGURE 5:**
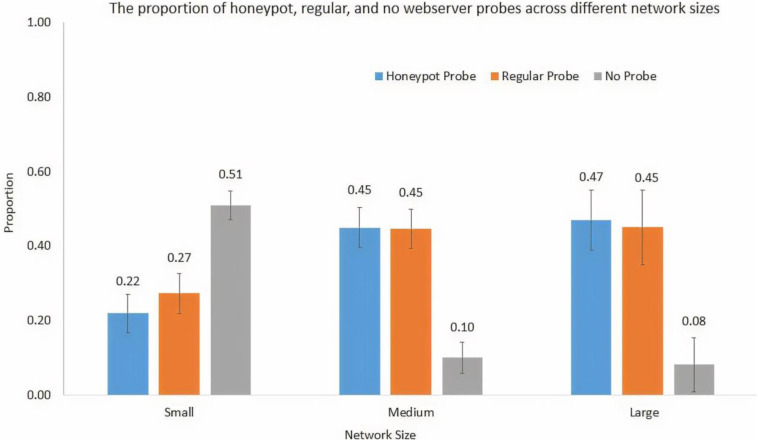
The proportion of honeypot probe, regular probe, and no-probe decisions across different network sizes.

We performed one-way ANOVA to investigate the influence of network size on decisions during the probe stage. The network size significantly influenced the proportion of honeypot web server probes [*F*(2,59) = 35.86, *p* < 0.001, η^2^ = 0.56], regular web server probes [*F*(2,59) = 18.31, *p* < 0.001, η^2^ = 0.39], and no web server probes [*F*(2,59) = 34.39, *p* < 0.001, η^2^ = 0.55], where *p*-value tests the statistical significance in the hypothesis test and η^2^ denotes the measure of the effect size. Figure 5 shows the proportion of honeypot, regular, and no web server probes across different network sizes.

As shown in Figure 5, the proportion of honeypot web server probes was 0.22 in the small network; however, the proportions of honeypot web server probes were 0.45 and 0.47 in the medium and large networks, respectively. The Tukey *post hoc* tests revealed that the proportion of honeypot web server probes in the small network was significantly smaller compared to the proportions of honeypot web server probes in the medium network (*p* < 0.001) and large network (*p* < 0.001). However, as per the Tukey *post hoc* tests, there were no significant differences between the proportions of honeypot web server probes in the medium and large networks (*p* = 0.83). These results are as per our expectations.

As shown in Figure 5, the proportion of regular web server probes was 0.27 in the small network; however, the proportions of regular web server probes were 0.45 and 0.45 in the medium and large networks, respectively. The Tukey *post hoc* tests revealed that the proportion of regular web server probes in the small network was significantly smaller compared to the proportions of regular web server probes in the medium network (*p* < 0.001) and large network (*p* < 0.001). However, as per the Tukey *post hoc* tests, there was no significant difference between the proportions of regular web server probes in the medium and large networks (*p* = 0.99). These results are as per our expectations.

As shown in Figure 5, the proportion of no web server probes was 0.51 in the small network; however, the proportions of no web server probes were 0.10 and 0.08 in the medium and large networks, respectively. The Tukey *post hoc* tests revealed that the proportion of no web server probes in the small network was significantly smaller compared to the proportions of no web server probes in the medium network (*p* < 0.001) and large network (*p* < 0.001). However, as per the Tukey *post hoc* tests, there was no significant difference between the proportions of no web server probes in the medium and large networks (*p* = 0.92). These results are as per our expectations.

### Influence of Network Size on Decisions During Attack Stage

**FIGURE 6:**
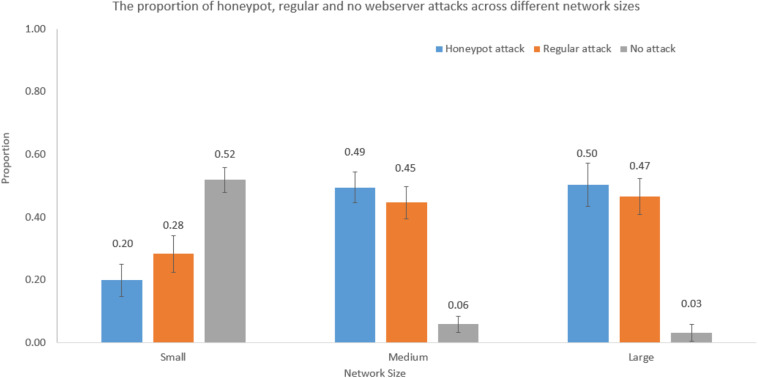
The proportion of honeypot attack, regular attack, and no-attack decisions across different network sizes.

We performed one-way ANOVAs to investigate the influence of network size on decisions during the attack stage. The network size significantly influenced the proportion of honeypot web server attacks [*F*(2,59) = 51.77, *p* < 0.001, η^2^ = 0.65], regular web server attacks [*F*(2,59) = 23.32, *p* < 0.001, η^2^ = 0.45], and no web server attacks [*F*(2,59) = 111.68, *p* < 0.001, η^2^ = 0.78]. Figure 6 shows the proportion of honeypot, regular, and no web server attacks across different network sizes.

As shown in Figure 6, the proportion of honeypot web server attacks was 0.20 in the small network; however, the proportions of honeypot web server attacks were 0.49 and 0.50 in the medium and large networks, respectively. The Tukey *post hoc* tests revealed that the proportion of honeypot web server attacks in the small network was significantly smaller compared to the proportions of honeypot web server attacks in the medium network (*p* < 0.001) and large network (*p* < 0.001). However, as per the Tukey *post hoc* tests, there were no significant differences between the proportion of honeypot web server attacks in the medium and large networks (*p* = 0.97). These results are as per our expectations.

As shown in Figure 6, the proportion of regular web server attacks was 0.28 in the small network; however, the proportions of regular web server attacks were 0.45 and 0.47 in the medium and large networks, respectively. The Tukey *post hoc* tests revealed that the proportion of regular web server attacks in the small network was significantly smaller compared to the proportions of regular web server attacks in the medium network (*p* < 0.001) and large network (*p* < 0.001). However, as per the Tukey *post hoc* tests, there was no significant difference between the proportion of regular web server attacks in the medium and large networks (*p* = 0.80). These results are as per our expectations.

As shown in Figure 6, the proportion of no web server attacks was 0.52 in the small network; however, the proportions of no web server attacks were 0.06 and 0.03 in the medium and large networks, respectively. The Tukey *post hoc* tests revealed that the proportion of no web server attacks in the small network was significantly smaller compared to the proportions of no web server attacks in the medium network (*p* < 0.001) and large network (*p* < 0.001). However, as per the Tukey *post hoc* tests, there was no significant difference between the proportion of no web server attacks in the medium and large networks (*p* = 0.73). These results are as per our expectations.

### Influence of Network Size and Sequential Probe/Attack Trials on Decisions

We performed mixed-factorial ANOVAs with network size as a between-subjects factor and sequential probe/attack trials as a within-subjects factor. The network size significantly interacted with sequential probe/attack trials for the following decisions: honeypot server probed and no server attacked [*F*(2,57) = 91.92, *p* < 0.001, η^2^ = 0.76]; regular server probed and honeypot server attacked [*F*(2,57) = 6.40, *p* < 0.001, η^2^ = 0.18]; regular server probed and no server attacked [*F*(2,57) = 81.23, *p* < 0.001, η^2^ = 0.74]; no server probed and regular server attacked [*F*(2,57) = 49.29, *p* < 0.001, η^2^ = 0.63]; no server probed and honeypot server attacked [*F*(2,57) = 54.15, *p* < 0.001, η^2^ = 0.66].

**FIGURE 7:**
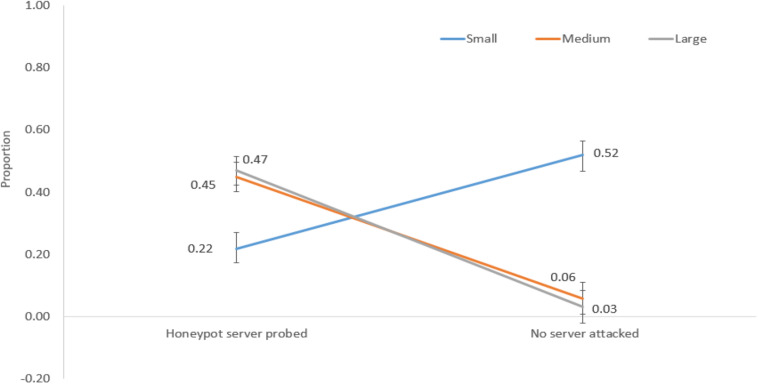
The proportions of honeypot server probed and no server attacked decisions in different network sizes.

Figure 7 shows the interaction between network size and honeypot server probed and no server attacked decisions. For a small network, the proportion of honeypot server probed was 0.22, and the proportion of no server attacked decisions was 0.52. However, for medium and large networks, the proportions of honeypot server probed were 0.45 and 0.47, and the proportions of no server attacked decisions were 0.06 and 0.03, respectively.

**FIGURE 8:**
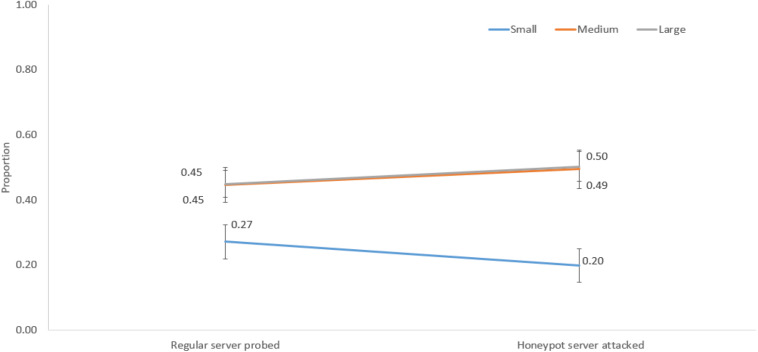
The proportion of regular server probed and honeypot server attacked decisions in different network sizes.

Figure 8 shows the interaction between network size and regular server probed and honeypot server attacked decisions. For a small network, the proportion of regular server probed was 0.27, and the proportion of honeypot server attacked decisions was 0.20. However, for medium and large networks, the proportions of regular server probed were 0.45 and 0.45, and the proportions of honeypot server attacked decisions were 0.49 and 0.50, respectively.

**FIGURE 9:**
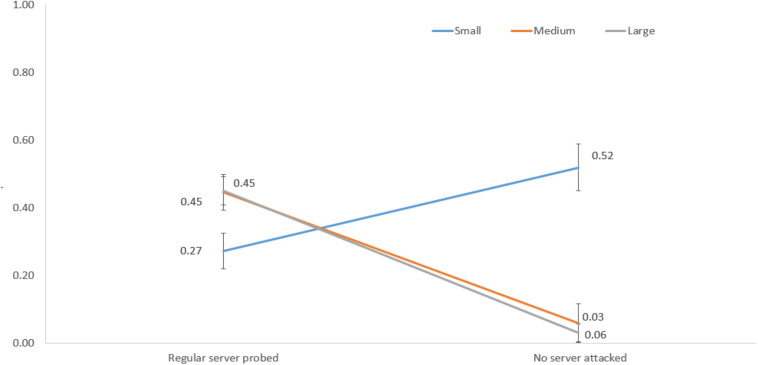
The proportions of regular server probed and no server attacked decisions in different network sizes.

Figure 9 shows the interaction between network size and regular server probed and no server attacked decisions. For a small network, the proportion of regular server probed was 0.27, and the proportion of no server attacked decisions was 0.52. However, for medium and large networks, the proportions of regular server probed were 0.45 and 0.45, and the proportions of no server attacked decisions were 0.06 and 0.03, respectively.

**FIGURE 10:**
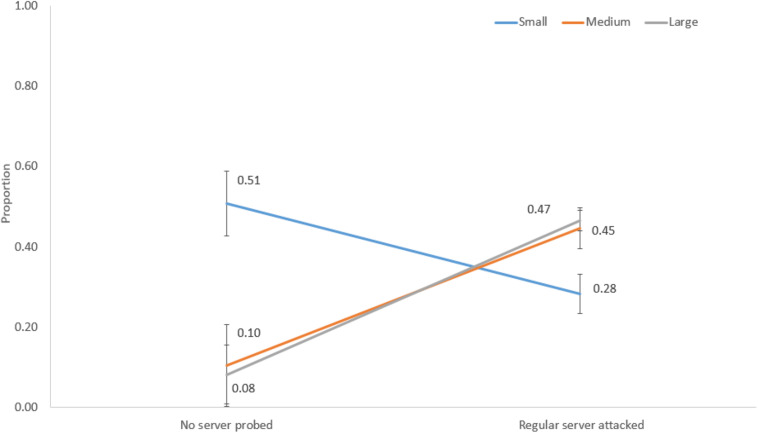
The proportions of no server probed and regular server attacked decisions in different network sizes.

Figure 10 shows the interaction between network size and no server probed and regular server attacked decisions. For a small network, the proportion of no server probed was 0.51, and the proportion of regular server attacked decisions was 0.28. However, for medium and large networks, the proportions of no server probed were 0.10 and 0.08, and the proportions of regular server attacked decisions were 0.45 and 0.47, respectively.

**FIGURE 11:**
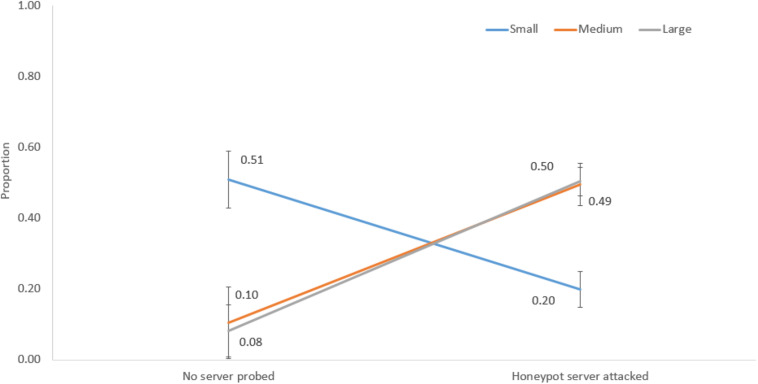
The proportions of no server probed and honeypot server attacked decisions in different network sizes.

Figure 11 shows the interaction between network size and no server probed and the honeypot server attacked decisions. For a small network, the proportion of no server probed was 0.51, and the proportion of honeypot server attacked decisions was 0.20. However, for medium and large network sizes, the proportions of no server probed were 0.10 and 0.08, and the proportions of honeypot server attacked decisions were 0.49 and 0.50, respectively.

## Discussion and Conclusion

Deception via honeypots can act as an essential tool to defend cyberattacks (Cohen, 2006; Rowe and Custy, 2007). Although prior research has developed and used games to understand the role of deception in cybersecurity, researchers had yet to investigate how the network’s size (i.e., the number of computers on the network) influences the adversary’s probe and attack decisions in the presence of deception via honeypots. To address this gap in the literature, in this paper, we investigated the influence of network size on adversary’s decisions in a DG involving honeypot web servers. Results revealed that the proportions of honeypot probe and attack actions and the proportions of regular probe and attack actions were more in medium- and large-sized networks compared to small-sized networks. Also, there was an influence of probing actions on attack actions across all three network sizes. These results can be explained based upon the IBLT, a theory of decisions from experience (Gonzalez et al., 2003; Gonzalez and Dutt, 2011, 2012; Dutt et al., 2013).

First, results revealed that the proportions of honeypot and regular probes and attacks were more in medium- and large-sized networks compared to small-sized networks. When the network size is small, the decisions during probe and attack stages in DG involve a choice between two web servers, where one of them is a honeypot. Given the smaller number of web servers, as per IBLT, it may be easier for bounded-rational participants to recall the mapping of web servers being regular or honeypot from memory. That is because about two instances are created in memory when there are two web servers, and the activation of these instances is likely to be much higher in memory due to smaller delays in their exploration during probing. However, in medium- and large-sized networks, due to the presence of multiple web servers, bounded-rational participants may not be able to easily recall the mapping of web servers as regular or honeypot from memory. That is because multiple instances, one per web server, would be created in memory, and the activation of these instances will likely decay in memory due to the long delays in their exploration during probing. Overall, as per IBLT, the difficulty in the recall of distant instances in medium- and large-sized networks may cause more exploration of web servers during the probe stage and the attack stage in these configurations compared to that in the small-sized network.

Second, the proportions of no-probe and no-attack actions were more in small-sized networks compared to medium- and large-sized networks. As per IBLT, a likely reason for these results is the differential activation of instances in memory for the no-probe and no-attack actions across the different-sized networks. As there would be fewer instances created in memory in small-sized networks compared to medium- and large-sized networks, these smaller numbers of memory instances corresponding to no-probe and no-attack actions are likely to be more activated in the small-sized network compared to medium- and large-sized networks. Overall, due to their higher activations, the no-probe and no-attack instances in memory will be easier to recall in a small-sized network compared to medium- or large-sized networks.

Third, we investigated the influence of network size and sequential probe/attack trials in DG. First, probing a honeypot caused an increase (decrease) in no server attacked actions in small (medium or large) networks. Second, probing a regular server caused a decrease (increase) in honeypot server attacked actions and an increase (decrease) in no server attack actions in small (medium or large) networks. Third, not probing a server caused a decrease (increase) in regular and honeypot server attacks in small (medium or large) networks. All these results can be explained based upon the differences in the activation and number of instances in memory in small-sized networks compared to large-sized networks. For example, as there were fewer and more activated instances likely created in memory of participants playing in small-sized networks compared to medium- and large-sized networks, the decisions of participants in small-sized networks were more logical and deterministic compared to those playing in medium- and large-sized networks. Due to these differences, perhaps, it was reasonable for participants playing in a small-sized network to show the above-stated results. At the same time, due to larger and weakly activated instances in memory of those playing medium- and large-sized networks, their decisions seemed to be less logical and more exploratory.

In this research, we performed a laboratory experiment using a canonical game, and our conclusions should be seen with this assumption in mind. However, our results have some important implications for the real world. First, our results reveal that making networks larger has an effect of increasing the proportion of regular probes and regular attacks. Thus, it may be advisable to break larger networks into smaller subnetworks, where these subnetworks may only possess a subset of computers (Achleitner et al., 2017). Furthermore, if these smaller subnetworks possess a number of honeypots, then these honeypots will likely cause adversaries to encounter them and not to attack the network. Also, a decrease in probes in these subnetworks may likely cause a decrease in the number of regular attacks.

One limitation of our research is that our results are derived from a lab-based experiment. It could be that the conditions stipulated in the lab are likely to be different from those simulated in the real world. However, as we tried to replicate the dynamics of cyberattacks in the DG game, i.e., search followed by an attack, some of the conclusions derived from our experiment are likely to be valid for the real world. Furthermore, the size of the networks chosen across different conditions in the experiment was done to investigate the effect of increasing the number of web servers. However, these network sizes are likely to be different from those encountered in the real world. There may be some networks where the number of web servers is in the range as those chosen by us in the experiment. For such networks, some of the conclusions in this study may be useful. Finally, motivated by the real world, we assumed that adversaries did not possess knowledge about what web servers were honeypots and whether deception was present in a particular round. If the presence of deception and honeypots is known to adversaries, then it is likely that adversaries may take advantage of this knowledge and end up attacking a larger proportion of regular web servers.

Currently, we investigated the influence of network size in DG, where the proportion of honeypots was kept constant in the game. Another possibility is to vary the proportion of honeypots in the game with different network sizes and evaluate the combined influence of these variations on adversarial probe and attack actions. A second possibility is to test how the variation in the cost of probes and attack actions influences these actions. Still, a third possibility is to test a team of adversaries playing in networks of different sizes and with different proportions of honeypots. Some of these ideas form the immediate next steps in our program on behavioral cybersecurity.

## Data Availability Statement

The datasets generated for this study are available on request to the corresponding author.

## Ethics Statement

The studies involving human participants were reviewed and approved by Ethics Committee, Indian Institute of Technology, Mandi. The patients/participants provided their written informed consent to participate in this study.

## Author Contributions

HK contributed to the design of the game, implementation of experimental protocols, and data collection. ZM contributed to the data analyses and development of models. VD and PA developed the idea of the study, and contributed to the design, implementation of the study, and writing of the manuscript. All authors contributed to the article and approved the submitted version.

## Conflict of Interest

The authors declare that the research was conducted in the absence of any commercial or financial relationships that could be construed as a potential conflict of interest.

## Supplementary Material

TABLE S1 Deception and non-deception rounds of deception game and instruction of deception game.

TABLE S2 Data collected for analysis.

## References

[B1] Achleitner S. La Porta T. F. McDaniel P. Sugrim S. Krishnamurthy S. V. Chadha R. (2017). Deceiving network reconnaissance using SDN-based virtual topologies. *IEEE Trans. Netw. Serv. Manag.* 14 1098–1112. 10.1109/tnsm.2017.2724239

[B2] Aggarwal P. Dutt V. (2020). The role of information about an opponent’s actions and intrusion detection alerts on cyber decisions in cyber security games. *Cyber Security* 3 363–378.

[B3] Aggarwal P. Gautam A. Agarwal V. Gonzalez C. Dutt V. (2019). “Hackit: a human-in-the-loop simulation tool for realistic cyber deception experiments,” in *Proceedings of the International Conference on Applied Human Factors and Ergonomics*, (Cham: Springer), 109–121. 10.1007/978-3-030-20488-4_11

[B4] Aggarwal P. Gonzalez C. Dutt V. (2016a). “Cyber-security: role of deception in cyber-attack detection,” in *Advances in Human Factors in Cybersecurity*, ed. Nicholson D. (Cham: Springer), 85–96. 10.1007/978-3-319-41932-9_8

[B5] Aggarwal P. Gonzalez C. Dutt V. (2016b). “Looking from the hacker’s perspective: role of deceptive strategies in cyber security,” in *Proceedings of the 2016 International Conference On Cyber Situational Awareness, Data Analytics And Assessment (CyberSA)*, (Piscataway, NJ: IEEE), 1–6.

[B6] Aggarwal P. Gonzalez C. Dutt V. (2017). “Modeling the effects of amount and timing of deception in simulated network scenarios,” in *Proceedings of the 2017 International Conference On Cyber Situational Awareness, Data Analytics And Assessment (Cyber SA)*, (Piscataway, NJ: IEEE), 1–7.

[B7] Aggarwal P. Gonzalez C. Dutt V. (2020). “HackIt: a real-time simulation tool for studying real-world cyberattacks in the laboratory,” in *Handbook of Computer Networks and Cyber Security*, eds Agrawal D. Perez G. M. Gupta D. Gupta B. B. (Cham: Springer), 949–959. 10.1007/978-3-030-22277-2_39

[B8] Aggarwal P. Moisan F. Gonzalez C. Dutt V. (2018). Understanding cyber situational awareness in a cyber security game involving recommendations. *Int. J. Cyber Situat. Awareness* 3 11–38. 10.22619/ijcsa.2018.100118

[B9] Almeshekah M. H. Spafford E. H. (2016). “Cyber security deception,” in *Cyber Deception*, eds Jajodia S. Subrahmanian V. Swarup V. Wang C. (Cham: Springer), 23–50. 10.1007/978-3-319-32699-3_2

[B10] Bace R. Mell P. (2001). *Special Publication on Intrusion Detection System.* Technical Report SP-800-31 Gaithersburg, MD: National Institute of Standards and Technology.

[B11] Bagchi K. K. Tang Z. (2004). Network size, deterrence effects and Internet attack incident growth. *J. Inform. Technol. Theory Appl.* 6:9.

[B12] Camerer C. F. (2003). *Behavioral Game Theory: Experiments in Strategic Interaction.* Princeton, NJ: Princeton University Press.

[B13] Cohen F. (2006). The use of deception techniques: honeypots and decoys. *Handb. Inform. Security* 3 646–655.

[B14] Cranford E. A. Lebiere C. Gonzalez C. Cooney S. Vayanos P. Tambe M. (2018). “Learning about cyber deception through simulations: predictions of human decision making with deceptive signals in stackelberg security games,” in *Proceedings of the 40th Annual Conference of the Cognitive Science Society*, At Madison, WI.

[B15] Dutt V. Ahn Y. S. Gonzalez C. (2013). Cyber situation awareness: modeling detection of cyber attacks with instance-based learning theory. *Hum. Fact.* 55 605–618. 10.1177/0018720812464045 23829034

[B16] Dutt V. Gonzalez C. (2012). Making instance-based learning theory usable and understandable: the instance-based learning tool. *Comput. Hum. Behav.* 28 1227–1240. 10.1016/j.chb.2012.02.006

[B17] Dutt V. Moisan F. Gonzalez C. (2016). “Role of intrusion-detection systems in cyber-attack detection,” in *Advances in Human Factors in Cybersecurity*, ed. Nicholson D. (Cham: Springer), 97–109. 10.1007/978-3-319-41932-9_9

[B18] Field A. (2013). *Discovering Statistics using IBM SPSS Statistics.* Thousand Oaks, CA: Sage.

[B19] Garcia-Teodoro P. Diaz-Verdejo J. Maciá-Fernández G. Vázquez E. (2009). Anomaly-based network intrusion detection: techniques, systems and challenges. *Comput. Security* 28 18–28. 10.1016/j.cose.2008.08.003

[B20] Garg N. Grosu D. (2007). “Deception in honeynets: a game-theoretic analysis,” in *Proceedings of the 2007 IEEE SMC Information Assurance and Security Workshop*, (Piscataway, NJ: IEEE), 107–113.

[B21] Gonzalez C. Dutt V. (2011). Instance-based learning: integrating sampling and repeated decisions from experience. *Psychol. Rev.* 118:523. 10.1037/a0024558 21806307

[B22] Gonzalez C. Dutt V. (2012). Refuting data aggregation arguments and how the instance-based learning model stands criticism: a reply to Hills and Hertwig (2012). *Psychol. Rev.* 119 893–898. 10.1037/a0029445

[B23] Gonzalez C. Lerch J. F. Lebiere C. (2003). Instance-based learning in dynamic decision making. *Cogn. Sci.* 27 591–635. 10.1207/s15516709cog2704_2

[B24] Heckman K. E. Walsh M. J. Stech F. J. O’boyle T. A. DiCato S. R. Herber A. F. (2013). Active cyber defense with denial and deception: a cyber-wargame experiment. *Comput. Security* 37 72–77. 10.1016/j.cose.2013.03.015

[B25] Hope C. (2020). *Cyber Losses Snowballing Despite an Increase in Cyber Security Spending.* Colorado Springs: Cyber Security.

[B26] Kiekintveld C. Lisý V. Píbil R. (2015). “Game-theoretic foundations for the strategic use of honeypots in network security,” in *Cyber Warfare*, eds Jajodia S. Shakarian P. Subrahmanian V. Swarup V. Wang C. (Cham: Springer), 81–101. 10.1007/978-3-319-14039-1_5

[B27] La Q. D. Quek T. Q. Lee J. Jin S. Zhu H. (2016). Deceptive attack and defense game in honeypot-enabled networks for the internet of things. *IEEE Internet Things J.* 3 1025–1035. 10.1109/jiot.2016.2547994

[B28] Lenin A. Willemson J. Sari D. P. (2014). “Attacker profiling in quantitative security assessment based on attack trees,” in *Proceedings of the Nordic Conference on Secure IT Systems*, (Cham: Springer), 199–212. 10.1007/978-3-319-11599-3_12

[B29] Mason W. Suri S. (2012). Conducting behavioral research on Amazon’s Mechanical Turk. *Behav. Res. Methods* 44 1–23. 10.3758/s13428-011-0124-6 21717266

[B30] Matthews K. (2019). *5 Futuristic Ways To Fight Cyber-Attacks.* Cologny: World Economic Forum.

[B31] Mell P. Hu V. Lippmann R. Haines J. Zissman M. (2003). *An Overview of Issues in Testing Intrusion Detection Systems.* Technical Report NIST IR 7007 Gaithersburg: National Institute of Standard and Technology.

[B32] PosTech (2020). *Attacks on Web Applications: 2018 in Review*. Available online at: https://www.ptsecurity.com/ww-en/analytics/web-application-attacks-2019/ (accessed July 10, 2020).

[B33] Rowe N. C. Custy E. J. (2007). “Deception in cyber attacks,” in *Cyber Warfare and Cyber Terrorism*, eds Janczewski L. Colarik A. (Pennsylvania: IGI Global), 91–96. 10.4018/978-1-59140-991-5.ch012

[B34] Sayegh E. (2020). *More cloud, More Hacks: 2020 Cyber Threats.* Available online at: https://www.forbes.com/sites/emilsayegh/2020/02/12/more-cloud-more-hacks-pt-2/#1868047e69b3 (accessed February 15, 2020).

[B35] Shang Y. (2018a). False positive and false negative effects on network attacks. *J. Stat. Phys.* 170 141–164. 10.1007/s10955-017-1923-7

[B36] Shang Y. (2018b). Hybrid consensus for averager–copier–voter networks with non-rational agents. *Chaos Solitons Fractals* 110 244–251. 10.1016/j.chaos.2018.03.037

[B37] Shang Y. (2019). Consensus of hybrid multi-agent systems with malicious nodes. *IEEE Trans. Circ. Syst. II Express Briefs* 67 685–689. 10.1109/tcsii.2019.2918752

[B38] Shimeall T. Spring J. (2013). *Introduction to Information Security: A Strategic-Based Approach.* London: Newnes.

[B39] Symantec (2019). *Symantec Internet Security Threat Report 2019.* Available online at: https://img03.en25.com/Web/Symantec/%7Bdfc1cc41-2049-4a71-8bd8-12141bea65fd%7D_ISTR_24_2019_en.pdf (accessed February 14, 2020).

[B40] Trustwave (2019). *Trustwave Global Security Report 2019.* Available online at: https://www.trustwave.com/en-us/resources/library/documents/2019-trustwave-global-security-report (accessed February 15, 2020).

[B41] Wang L. Jajodia S. Singhal A. Noel S. (2010). “k-zero day safety: measuring the security risk of networks against unknown attacks,” in *Proceedings of the European Symposium on Research in Computer Security*, (Berlin: Springer), 573–587. 10.1007/978-3-642-15497-3_35

