# Supplementary material for: Influence of Network Size on Adversarial Decisions in a Deception Game Involving Honeypots
Source: Front Psychol. 2020 Sep 25;11:535803. doi: 10.3389/fpsyg.2020.535803 (PMC7575868; doi:10.3389/fpsyg.2020.535803)
Supplement: TABLE S1 — Deception and non-deception rounds of deception game and instruction of deception game. [file Table_1.DOCX]

**Supplementary Material**

Table S1 shows the deception and non-deception rounds in the DG game across 29-rounds. Each round contained a probe stage and an attack stage.

Table S1. Presence of deception and non-deception rounds in the deception game.

| Round Number | Deception/Non-deception |
| --- | --- |
| 1 | D |
| 2 | N |
| 3 | D |
| 4 | D |
| 5 | N |
| 6 | N |
| 7 | D |
| 8 | D |
| 9 | D |
| 10 | D |
| 11 | D |
| 12 | N |
| 13 | N |
| 14 | N |
| 15 | D |
| 16 | N |
| 17 | N |
| 18 | N |
| 19 | N |
| 20 | D |
| 21 | D |
| 22 | N |
| 23 | D |
| 24 | N |
| 25 | D |
| 26 | N |
| 27 | N |
| 28 | D |
| 29 | D |

**Instruction of deception game with six web servers**

Welcome! This study consists of a single task, where you will be playing as a hacker against a computer system. Hackers are people knowledgeable about computers and they use this knowledge to steal data and private information in networks and damage systems that are important for organization.

A webserver is a computer that enables customers on the Internet to interact with a website. Shoppers.com, an online retail website, has setup six webservers. Some of these webservers are called regular, and these regular webservers store data about the customers, transaction information, and customers' personal information including preferences for products. The other webservers are called honeypot, and these webservers pretend to be regular webservers. Actually, the honeypot webservers are fake, and their main function is to trap you when you try to attack the network.

In this task, webservers in the network will appear as six buttons labeled as webserver 1, webserver 2, ..., webserver 5, and webserver 6 on a computer screen. In order to attack Shoppers.com, you are allowed to probe any of the six webservers multiple times (probe stage). In the probe stage, you can probe up to three webservers, or you may not probe any webserver. Probing means that you try to collect information on whether a webserver is a honeypot or a regular webserver. To probe a webserver, you need to click on the button corresponding to the webserver once. In response to your action, the network would reply with a “regular” or a “honeypot” message. You may trust this information; however, the network response could be false and you may be deceived by the network.

Once you have probed the network, you will enter the attack stage. In the attack stage, you may decide either to attack one of the six webservers for real or to not attack any webserver. In the attack stage, a webserver could be attacked for real by clicking the button of the webserver that you want to attack.

There will be multiple rounds in this game, where each round will consist of a probe stage and an attack stage on a new set of six webservers. You may win or lose points during the probe stage and the attack stage based on whether you were able to probe and attack a regular or honeypot webserver. Payoffs at both the stages are represented in table below:

| Your Action | Your Payoff |
| --- | --- |
| Probe a Regular Webserver | 5 points |
| Probe a Honeypot Webserver | -5 points |
| Decide not to probe the network | 0 points |
| Attack a Regular Webserver | 10 points |
| Attack a Honeypot Webserver | -10 points |
| Decide not to attack the network | 0 points |

You will be given INR 50 for your participation and your goal in this task is to win as many points as possible. At the end of the game, we will randomly select 3 top scoring Hacker participants based upon total points won in the game. These 3 Hacker participants will enter a lucky draw. In the lucky draw, 1 Hacker participant will be randomly selected and given an Amazon.in's gift voucher of INR 500 denomination as a prize.
